# An Unique Case of Dislocation of the Patella.—Complete Revolution of the Bone on Its Long Axis

**Published:** 1856-06

**Authors:** 


*An Unique Case of Dislocation of the Patella—Complete Revolution of the Bone on its long Axis.*

On 29th June, 1855, a black man was engaged in loading lumber upon a boat. It was conveyed by means of a car running on rails. He carelessly allowed himself to be caught by the loaded car, while it was in motion, and jammed against the cross sticks on which the piles of lumber rested. The accident occurred about 5 o'clock, P. M., and I saw him in half an hour. He was lying on his back, and his right leg was stretched stiffly out. The knee was entirely changed in form. A sharp and prominent edge was apparent on the outer part of the joint, projecting about half an inch forward of the normal plane of the anterior face of the patella, and about the same distance over the outer edge of the joint. Beneath this edge there was a depression into which the ends of the fingers could be readily passed, and at the bottom of which the outer edge of the articulating surface of the outer condyle of the femur could be felt. The inner part of the joint was flattened and depressed, and the fingers could readily detect the inner edge of the inner condyle of the femur over which no portion of the patella rested. Passing the fingers from this point outwards, and pressing well upon the surface of the bone, a depressed edge was plainly felt, about half an inch or less from the rough elevation which borders the articulating surface of the inner condyle of the femur, on its inner edge. Above and below the patella, an evident twist was discovered in the tendinous insertion of the extensor muscles, and in the ligament of the patella. The joint was stiff, and allowed but limited motion.

From these appearances I recognized a dislocation of the patella, by which the bone was twisted completely round on its longitudinal axis, so that its outer edge corresponded, nearly, to the inner edge of the articulating surface of the femur: its anterior face was turned backwards, and rested on the same articulating surface; its inner edge looked outwards and a little forwards, forming the projecting edge in front and on the outside of the joint; and its posterior or articulating face was under the skin, looking forwards with a slight inclination inwards.

To remedy this displacement the indication was evident. It was, to untwist the part. But before attempting anything, it was necessary to determine the exact nature of the twist, whether in coming to its new position the projecting (inner) edge of the bone had passed forwards and then outwards, or backwards and outwards. An inspection of the parts, and a consideration of the manner in which the accident had happened, showed that the former was the true mechanism of the dislocation. The inspection showed this by the evident direction in the twist of the ligaments; and the manner in which the accident occurred, proved the same state of things. Thus: the man was standing in front of the car, so placed against the pile of boards, that his thigh was firmly fixed on its inner side, the outer and anterior part of the leg presenting to the approaching car. The projecting edge of the car, or the end of one of the boards with which it was loaded, struck the outer edge of the patella; and as the car moved on, (the limb being firmly fixed,) the force applied to the bone on its outer edge, acted in such a way as to press this edge backwards and inwards, causing it to slip along the large articulating face of the external condyle, while the inner edge of the patella, following a converse direction, was tilted forwards. Had the force ceased to act at this point, there would have been a dislocation to the extent of that already described by several authors, only that instead of the outer edge of the patella presenting forwards, the inner edge backwards, the posterior or articulating face outwards, and the anterior face inwards, the positions would have been just the reverse. But as the force did not cease to act, nor the bone to yield to it, the outer edge of the patella was driven onwards, and made to mount the small articulating face of the inner condyle. Here it rested; and as the inner edge of the bone was performing a counter movement under the skin, the result was a complete revolution of the patella on its long axis.

An inspection of the two bones concerned in this dislocation will show that when their relative positions have been changed in the manner and to the extent described, the appearances given to the joint must necessarily be such as were seen in this case. The articulating surfaces of the bones are both divided into two parts: that of the patella by an elevated ridge, and that of the femur by a deep depression. In both the bones the outer of these surfaces are large, the inner small; the outer surface of the patella being larger than that of the femur, so that the outer edge of the former bone overlaps the outer edge of the latter. From this arrangement of the parts it follows that when the patella is displaced in the manner now under consideration, its inner border must project slightly beyond the outer border of the external condyle of the femur, and thus give rise to one of the most striking symptoms of the luxation, viz: the well defined prominence which was given to the outer part of the knee joint in the case before us, and also to other symptoms discribed by authors, viz: the perceptible ridge under the skin due to the ridge on the posterior face of the patella.

Again: of the two surfaces of the femur the outer is much the most prominent, while of the two edges of the patella the inner is much the thicker; hence in the new position of the bone an unnatural prominence must be given to the outer part of the joint. This alteration in the shape of the knee was another striking symptom in the dislocation.

From a consideration of all these points there could be no doubt that the bone had come into its new position by an entire revolution, in which its outer edge (which received the force) revolved from before backwards, and from without inwards, the inner edge performing a counter movement in correspondence with that of the outer. In a thin skined subject there will probably be no difficulty in discriminating the surface which is presenting under the skin by the prominent ridge which divides the articulating faces; for if this be detected with the fingers, it must remove all uncertainty as to which face of the bones is forward.

In looking at the authorities on this subject, I find that the accident was not recognised by surgeons of comparatively recent dates. Sir Astley Cooper, and writers of his day, were only acquainted with three kinds—outwards, inwards and upwards. In Bransby Cooper's edition of Sir Astley's work on Dislocations, published in this country in 1844, he gives the old enumeration, but in the list of difficult cases describes one seen by Mr. Welling, "in which the patella was dislocated upon its edge. The nature of the accident was obvious, as the edge of the bone forced up the integuments to a considerable height between the condyles on the fore part of the joint. It was reduced with difficulty by pressing the edges of the bone in opposite directions when the leg was extended."

The same authority furnishes another case from Mr. Mayo. Two lines of cavalry were riding through each other on exercise, when the knees of two horsemen struck. "The patella," it is said, "was dislocated outwards, and rested with its inner edge upon the outer surface of the external condyle, the fore part of the patella facing forwards and inwards." There was great difficulty in reducing it, and it was finally done by suddenly carrying the heel back to the hip.

The same authority quotes from the London Medical Gazette, p., 320, as taken from Rust's Magazine, the case of a huzzar riding without stirrups. His horse started and struck his left leg against his comrade. "The bone had undergone a *semi-revolution in its longitudinal axis;* its inner edge rested fixed in the trochlea, between the condyles of the femur; the outer projected directly forwards; the anterior surface was directed inwards and the posterior outwards." It was detected by the ridge on the posterior face of the patella and by "the direction inwards of the very tense tendo-communis-extensorum-cruris and the ligament of the patella." Attempts at reduction failed, and so did the extraordinary operation which was undertaken of cutting down upon the bone, severing the common ligament, and then the ligament of patella, and then trying to replace the bone.

The Dictionaire de Médecine describes what is there called vertical luxations. They are said to have been mentioned by Moscati, Manne and Léveillé, though the first authentic case is related by Abbondio Gelodi in 1750. They were not, however, recognised by writers as late even as Boyer, Delpech, Samuel or Astley Cooper.

Two kinds are described, the internal vertical, and the external vertical. "In the first, the patella being pushed inwards, has its anterior face looking outwards, the posterior face inwards: the inner border is anterior and the outer border posterior."

"In the external vertical the patella pushed outwards is placed with its internal border on the femur, and its external border under the skin."

This last, it will be seen, corresponds with that of the case from Rush.

Vidal de Cassis, in his "Traité de Pathologie Externe et de Médecine Opératoire," describes the vertical luxation where the bone, "turning on its axis, becomes fixed vertically; when one of its borders becomes entirely anterior, the other posterior, the two faces looking to the two sides."

A remark of importance to my case occurs in this author, in describing the luxations of the patella, and after what has just been quoted, he says, (vol. 2, p. 354,) "Can a luxation of the patella exist which shall consist in a complete rotation on the great axis of this bone, and which shall place its cartillaginous face in front, its sub-cutaneous behind? Such a reverse ment," he continues, "supposes such disorder in the articulation, that the fact of the luxation would be, perhaps, the least important element in the injury of the knee."

The case I am describing will prove not only that this complete revolution is possible, but that it can occur without any injury to the joint, and is also susceptible of easy reduction.

Pirrie, in his System of Surgery, recognises the semi-revolution of the patella, describing it as above, and giving the three cases already quoted. Erchsen says: "In some cases the bone is turned almost completely round, the posterior surface becoming partly anterior." This comes nearer the case I am describing than anything yet brought forward; but as no case is given, we are left in ignorance as to whether it is only what the writer supposes may occur, or what he has seen. "If the relaxation of the muscles of the thigh and the employment of proper pressure upon the patella do not succeed, reduction," Erichsen says, "may perhaps be effected by directing the patient to make a sudden and violent muscular effort at extension of the limb, or by attempting to walk. In other cases, again, it may be readily replaced by bending the leg and rotating it on the axis of the tibia at the same time that the patella is pressed into position, as Vincent recommends. Should these plans not answer, I do not think it would be advisable to have recourse to sub-cutaneous section of the tendon of the quadriceps extensor and the ligamentum patella."

Malgaigne, in his Traité des Fractures et des Luxations, very recently published, gives the only case I have been able to find which resembles the one I have described, if indeed it was quite as complete. It occurred in a way not unlike mine; and it is perhaps to be taken as an example of the same kind, even though the rotation may not have been quite as complete, or the articulating surface of the femur as much covered.

With regard to that point, I would remark, that by placing the bones in "situ," and then giving them the motion by which this dislocation is produced, it will be seen that the exact extent of the rotation may vary, and in one case the posterior face of the patella be brought more completely forward than in another, while the mechanism of the displacement being exactly the same, it may seem unnecessary to divide them into two classes.

The case was that of "a girl 17 years old, who, bending forward to take a book from a table, placed the whole weight of the body on the extended right leg, resting the outer edge of the patella on the edge of a chair, when she suddenly cried out: the patella was luxated. M. Castara, immediately called, found the leg half bent, and capable of but little extension. The patella, resting by its external border on the outer and upper side of the articular face of the femur, of which it covered only the size of 6 or 8 millimètres, had its inner border inclined outwards, and forming a projection in this direction of 2½ centimètres, its articular face looking forwards and inwards. The tendon and ligament of the patella formed a rounded cord above and below, tolerably thick and hard. The surgeon seized the patella with the thumb and index, and by a simple movement of rotation from behind forwards, and from without inwards, brought it easily to its place."

There was evidently more external dislocation here than in my case. The patella did not lie so completely in front of the articulating surface of the femur, being on the "outer and upper side," nor was its articular face looking so completely forward.

The reduction was effected without difficulty. The thumbs of both hands being placed on the outer and under edge of the projecting border of the patella, while the index and middle fingers were pressed against the other border in a direction outwards and backwards, force was applied with a view to roll the bone over into its place. The first efforts failing, a by-stander was directed to pass his hands under the knee joint, and make forcible and intermittent flexion of the leg. In a moment the bone performed its evolution, slipped into its place, and the man rose up and walked. He experienced no further inconvenience, the ligaments, carlages, and investing membranes of the joint having received no injury whatever from this extensive displacement.—*Chas. Med. Journal.*

